# Extending World Health Organization weight-for-age reference curves to older children

**DOI:** 10.1186/1471-2431-14-32

**Published:** 2014-02-03

**Authors:** Celia Rodd, Daniel L Metzger, Atul Sharma

**Affiliations:** 1 Section of Pediatric Endocrinology, Children’s Hospital of Winnipeg, FW 302-685 William Ave, Winnipeg, MB R3E 0Z2, Canada; 2 Division of Pediatric Endocrinology, BC Children’s Hospital, 4480 Oak Street, Vancouver, BC V6H 3V4, Canada; 3 Department of Pediatrics, Children’s Hospital of Winnipeg, 840 Sherbrook Street, Winnipeg, MB R3A 1S1, Canada; 4 Biostatistical Consulting Unit, George and Fay Yee Center for Healthcare Innovation, University of Manitoba, GH706-820 Sherbrook Street, Winnipeg, MB R3A1R9, Canada

**Keywords:** Growth, Growth charts, Anthropometry, Pediatrics, Child

## Abstract

**Background:**

For ages 5–19 years, the World Health Organization (WHO) publishes *reference* charts based on ‘core data’ from the US National Center for Health Statistics (NCHS), collected from 1963–75 on 22,917 US children. To promote the use of body mass index in older children, weight-for-age was omitted after age 10. Health providers have subsequently expressed concerns about this omission and the selection of centiles. We therefore sought to extend weight-for-age reference curves from 10 to 19 years by applying WHO exclusion criteria and curve fitting methods to the core NCHS data and to revise the choice of displayed centiles.

**Methods:**

WHO analysts first excluded ~ 3% of their reference population in order to achieve a “non-obese sample with equal height”. Based on these exclusion criteria, 314 girls and 304 boys were first omitted for ‘unhealthy’ weights-for-height. By applying WHO global deviance and information criteria, optimal Box-Cox power exponential models were used to fit smoothed weight-for-age centiles. Bootstrap resampling was used to assess the precision of centile estimates. For all charts, additional centiles were included in the healthy range (3 to 97%), and the more extreme WHO centiles 0.1 and 99.9% were dropped.

**Results:**

In addition to weight-for-age beyond 10 years, our charts provide more granularity in the centiles in the healthy range −2 to +2 SD (3–97%). For both weight and BMI, the bootstrap confidence intervals for the 99.9th centile were at least an order of magnitude wider than the corresponding 50th centile values.

**Conclusions:**

These charts complement existing WHO charts by allowing weight-for-age to be plotted concurrently with height in older children. All modifications followed strict WHO methodology and utilized the same core data from the US NCHS. The additional centiles permit a more precise assessment of normal growth and earlier detection of aberrant growth as it crosses centiles. Elimination of extreme centiles reduces the risk of misclassification. A complete set of charts is available at the CPEG web site (http://cpeg-gcep.net).

## Background

Growth charts are a graphic representation of anthropometry in a population and are critical to health-care providers assessing patterns of growth, body shape, and size [1,2]. Calculated z-scores for body mass index (BMI), height, and weight are also important research tools [1].

The World Health Organization (WHO) has published *standard* curves for children aged 0–5 years (y) based on their Multicentre Growth Reference Study (MGRS); these charts are intended to reflect growth under ‘optimal conditions’ [3,4]. WHO also published *reference* curves (‘how children currently grow’) for children 5–19y based on ‘core data’ from the US National Center for Health Statistics (NCHS) collected from 1963–1975 [5]. This was the same dataset used in the construction of the original *1977 NCHS Growth Charts*[6] and was a key source for the Centers for Disease Control and Prevention (CDC) 2000 revision [7,8].

North American adoption of the WHO charts has been variable. In the US, the CDC now recommends the use of the *WHO Child Growth Standards* for children 0–2y of age and the *CDC Growth Charts* for older children [9]. In Canada, the Dietitians of Canada, the Canadian Paediatric Society, the Public Health Agency of Canada and others have endorsed both sets of WHO charts in the form of the ‘2010 WHO Growth Charts for Canada’ [2]. Nevertheless, a 2011 position paper from the Canadian Pediatric Endocrine Group (CPEG) highlighted practical concerns from their membership and the general pediatric community, including the lack of weight-for-age reference curves beyond age 10y [10,11]. A recent survey of chart users by the Canadian Paediatric Society also highlighted practical obstacles to routine application of the WHO charts, including the lack of weight-for-age beyond age 10y and the sparseness of the centile lines in the normal range. Users also commented on the potentially misleading inclusion of centiles 0.1 and 99.9 (±3 SD) [12]. Omission of weight-for-age curves beyond age 10y prompted the Dietitians of Canada and the Public Health Agency of Canada to explore remedies with the WHO, but lack of resources precluded the required re-analysis [13].

The primary objective of this study was to develop complementary charts extending weight-for-age for ages >10y through strict application of WHO exclusion criteria and curve-fitting methods to the core NCHS data. The secondary objectives were to evaluate the precision of these centiles through bootstrap resampling and to prepare charts with a more useful selection of growth centiles in the normal range.

## Methods

### Statistical analyses

#### Extension of weight-for-age

WHO reference curves are based on ‘core data’ from the US NCHS, collected from 1963–1975 on 11,507 girls and 11,410 boys aged 1–24y, kindly provided by Dr. M. de Onis of the WHO [5]. A waiver for use of this anonymized, publically available dataset was obtained from the Institutional Review Board of the Montreal Children’s Hospital (McGill University). For the development of WHO reference curves for school-aged and adolescent children, these data were merged with ~8,000 cross-sectional observations from the MGRS (ages 18–71 months) to smooth the transition at age 5y [5]. These MGRS data are not yet in the public domain.

To extend the weight-for-age reference curve beyond age 10y, WHO exclusion criteria were first applied to create a reduced dataset (NCHS-R) with 11,193 girls and 11,106 boys. As in the WHO reports, there were exclusions for both ‘outlying’ heights-for-age (14 girls, 8 boys) and ‘unhealthy’ weights-for-height (300 girls, 296 boys), the latter defined by the WHO as weights-for-height <0.135^th^ or >97.7^th^ centiles (−3 and +2 SD, respectively). After exclusions, there were 673 ± 204 (mean ± SD) boys and 646 ± 185 girls for each annual interval between 5-19y. Detailed descriptive statistics for each cohort are summarized in (Additional file 1: Table S1). WHO global deviance and information criteria [3-5] were then applied using the GAMLSS statistical package of Stanisopoulos and Rigsby to develop optimal Box-Cox power exponential (BCPE) models that explicitly fit the time-evolution of 4 parameters: μ (median), σ (coefficient of variation), ν (skew) and τ (kurtosis) [14,15]. These parameters are then joined by cubic splines with degrees of freedom (df) chosen to balance accuracy and smoothness. Before the BCPE model was applied, the time axis also required a power transformation (exponent λ) to better capture periods of rapid change [4,5,14,16]. A detailed review of the exclusion process, modeling procedure, and diagnostic validation is found in the Statistical Methods and Models manual at the CPEG website [17]. Optimal models were

● For girls, λ = 1.22, df(μ) = 14, df(σ) = 6, df(ν) = 5, and τ = 2

● For boys, λ = 1.30, df(μ) = 13, df(σ) = 8, df(ν) = 5, and τ = 2

When τ = 2, kurtosis may be ignored, and the BCPE model reduces to the simpler 3-parameter skew normal or LMS model (L = ν, M = μ, and S = σ) [4,5,14,16]. Model fit was confirmed through standard goodness-of-fit tests and diagnostic plots [4,8,17].

For specific ages and genders, published LMS data (WHO reference and standard) were used to generate smoothed centiles 3, 10, 25, 50, 75, 90, and 97 (−2 to +2 SD) for height-for-age, BMI-for-age (2–19y), length-for-age, length-for-weight, and head circumference-for-age (0–2y). The same is true for the weight-for-age curves from 2–10y. Beyond age 10y, weight-for-age centiles are based on the NCHS-R dataset fitted here.

#### Comparison with ‘2010 WHO growth charts for Canada’

At monthly intervals from 5–10 years of age, calculated weight-for-age centiles were compared to corresponding WHO centiles using the absolute deviation in kilograms (kg, mean ± SD).

#### Comparing smoothed to empiric centiles (NCHS-R)

For ages 5–19y, the smoothed centile lines calculated from their LMS parameters were compared graphically to empiric centiles, which were calculated separately for each gender after the raw NCHS-R data were grouped (binned) by year of age. In addition, the smoothed centiles were used to determine the proportion of the reference population falling below each centile line.

#### Bootstrap resampling

To estimate sample bias, standard errors, and 95% confidence intervals, smoothed LMS centile curves for each gender and anthropometric measure were fitted to 1,000 nonparametric bootstrap replicates drawn from the NCHS data. The 95% confidence intervals are the standard intervals of Efron and Tibshirani [18,19]. The same procedure was used to examine the effects of sample size as bootstrap samples were varied from 50–300 per month (total sample 10,250–61,500).

All statistical analyses were performed in R [20]. Unless otherwise noted, all values are means ± standard deviation (SD) and statistical significance is p <0.05.

## Results

**Figure 1:**
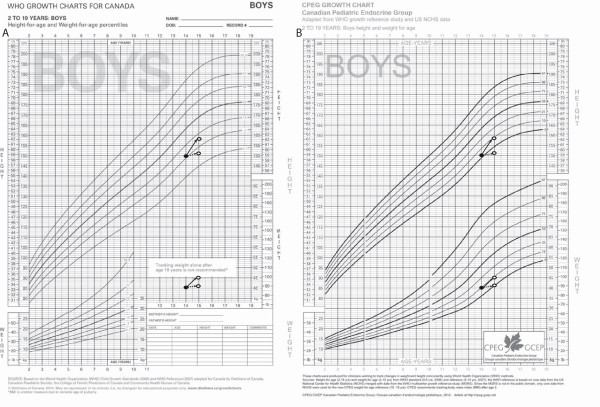
**2010 WHO Growth Charts for Canada and CPEG Growth Charts for Canada. A)** ‘2010 WHO Growth Charts for Canada’, height and weight for boys aged 2–19y. Weight-for-age centiles 0.1, 3, 15, 50, 85, and 97 span ages 2–10y. Reprinted with permission. **B)** CPEG growth charts, height and weight for boys aged 2–19y: After age 10y, weight-for-age centiles 3, 10, 25, 50, 75, 90, and 97 are based on re-analysis of the NCHS-R dataset. To illustrate difficulties in the interpretation of short-term growth changes without a weight-for-age reference, we plot two alternate growth trajectories, identified by solid and dashed arrows. Under both scenarios, the BMI is 18.0 kg/m^2^ at both the beginning (closed circles) and end (open circles) of the observation period. It is clear that the weight-for-age reference facilitates interpretation of the different trajectories.

In Figure 1B, we see an example of the CPEG growth charts, intended to complement existing ‘2010 WHO Growth Charts for Canada’ (Figure 1A). The CPEG chart depicts height and weight for boys aged 2–19y with centiles 3, 10, 25, 50, 75, 90, and 97 (−2 to +2 SD). For ages 2–10y, height-for age and weight-for-age curves were generated from published LMS data (WHO standard and reference). Beyond age 10y, weight-for-age centiles are based on the NCHS-R dataset fitted here.

**Figure 2:**
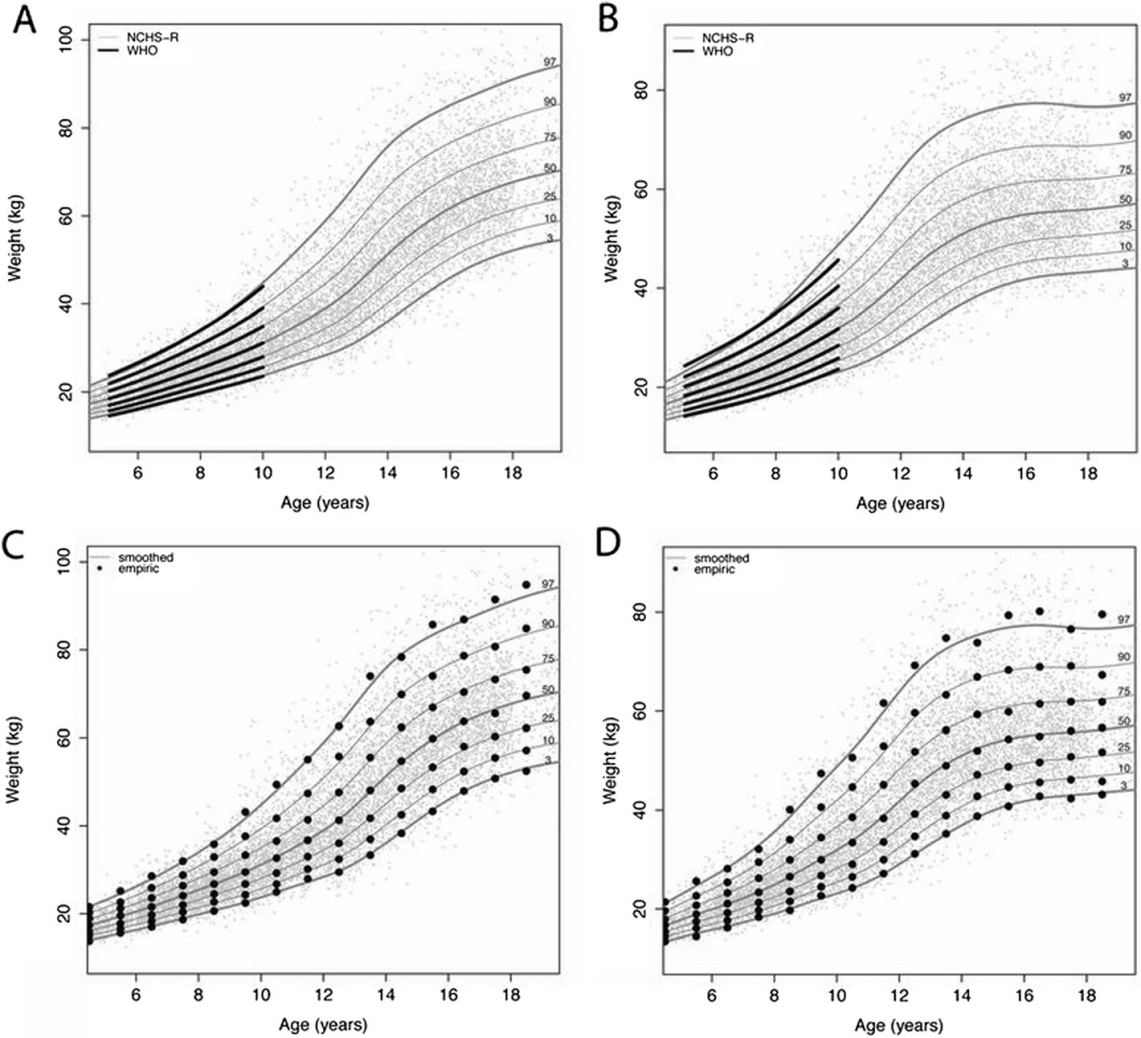
**Comparison of smoothed and empiric centiles: figures A (boys) and B (girls) compare WHO smoothed centiles with those calculated from the NCHS-R dataset.** For the latter, smoothed and empiric centiles (data binned by year of age) are compared for boys **(C)** and girls **(D)**. The individual gray points depict raw data.

To assess agreement between CPEG and WHO weight-for-age curves, the 2 charts were first compared graphically. The smoothed weight-for-age reference curves from the core NCHS data (NCHS-R) span ages 5–19y and overlap with WHO curves for ages 5–10y for boys (Figure 2A) and girls (Figure 2B). For boys, all centiles from 3–97 align closely in the overlap zone (5–10y). For girls, this was also true for the median and for centiles below the median. There is, however, disagreement at higher centiles, particularly the 97^th^, with weights tending to be higher in the North American cohort (i.e. rightward skew). These differences are quantified in Table 1, which displays the absolute discrepancy in kg (mean ± SD) between NCHS-R and WHO smoothed centiles based on monthly estimates from 5–10y. The results mirror those presented graphically.

**Table 1:** Mean absolute discrepancy (MAD, kg)

**Smoothed centiles**	**MAD (boys)**	**MAD (girls)**	**% below (boys)**	**% below (girls)**
3	0.12 ± 0.07	0.20 ± 0.11	2.9	2.9
25	0.05 ± 0.03	0.07 ± 0.05	24.5	25.1
50	0.02 ± 0.02	0.17 ± 0.07	50.3	50.1
75	0.08 ± 0.06	0.37 ± 0.18	75.6	75.5
97	0.37 ± 0.21	1.18 ± 0.91	96.8	96.7

Mean absolute discrepancy (MAD, kg ± SD) is based on monthly observations from NCHS-R and WHO weight-for-age reference curves (5–10y). The % below refers to the proportion of reference population (NCHS-R raw data) below the smoothed centile curves, ages 5–19y.

While less germane, our extension to the WHO weight-for-age reference can also be compared directly to CDC 2000 centiles as in Table 1 (Additional file 1: Table S2). These differences are consistent with what has previously been described in formal comparisons of CDC 2000 and WHO references charts [21], reflecting significantly different reference populations and exclusion criteria, since the CDC included North American survey data through 1994 and the 3% of children with ‘unhealthy’ weights-for-height by WHO criteria [5,7,8]. These curves were further validated through comparison of smoothed and empiric centiles, the latter based on the same raw data binned by year of age. Figures 2C (boys) and 2D (girls) show good agreement between smoothed and empiric centiles for all ages 5–19y. In addition, Table 1 displays the proportion of the raw data falling below each centile line, confirming that the smoothed centiles effectively capture the empiric centiles in the reference population.

**Figure 3:**
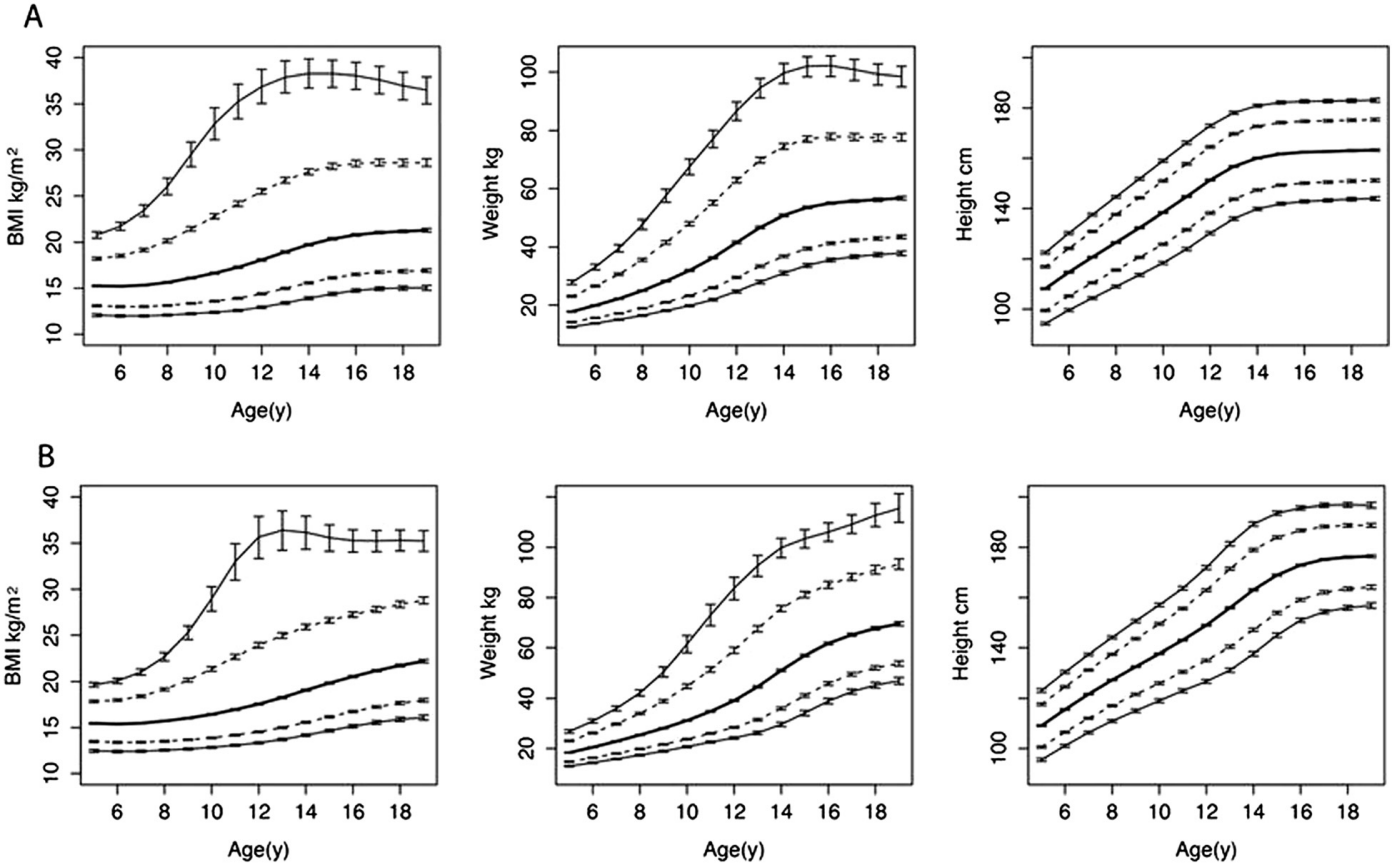
**Bootstrap estimates of centile precision: smoothed centiles for girls (A) BMI, weight, and height were first fitted to the original NCHS-R data (N = 11,193).** The 95% confidence intervals (CI) were calculated from 1,000 bootstrap samples drawn from the original data with replacement and analyzed in the same way. Each figure displays centiles 0.135, 3, 50, 97, and 99.9, with error bars to denote the corresponding 95% CI at yearly intervals from 5–19y. Similarly, boys **(B)** BMI, weight, and height centiles were fitted to data on 11,106 boys and CI calculated from the analysis of 1,000 bootstrap replicates.

Bootstrap replicates were studied to better understand the accuracy and precision of the fitted centiles. For all physical measures, centiles 0.135–97 (−3 to +2SD) were unbiased and narrowly estimated (Figure 3). However, for both weight and BMI, the confidence intervals for the 99.9^th^ centile (+3SD) were at least an order of magnitude wider than the corresponding 50^th^ centile values. In 10-year-old girls, for example, the 95% confidence interval for the 99.9^th^ centile ranged from 31.1–34.6 kg/m^2^ (BMI) and 64.8–70.2 kg (weight). To achieve precision comparable to the other curves, the total sample size would need to be approximately 60,000 subjects of each gender. These results lead to the creation of charts ranging from –2SD to + 2SD (3–97%), with additional centile lines in the healthy range and weight-for-age extension beyond age 10y (Figure 1). The example in Figure 1 highlights the importance of concurrent weight-for-age plots for the correct interpretation of short-term growth changes, since BMI is the same in both of the illustrated growth trajectories.

## Discussion

The 2006 WHO growth *standard* (0–5y) is based on the MGRS, which examined 8,440 breastfed infants and children from 6 countries. The MGRS combined a longitudinal follow-up and a cross-sectional survey of children; families were carefully selected to ensure conditions favoring achievement of their full genetic growth potential [3]. In contrast, the core data for the 2007 WHO growth *reference* derive from a historical North American cohort [5] previously used to develop the *1977 NCHS Growth Charts*[6] and their 2000 CDC revision [7,8]. To smooth the transition between the WHO standard and reference charts at age 5, these core NCHS data were merged with cross-sectional observations from the MGRS (ages 18–71 months) [5]. The data were further selected through exclusion of ~3% of the population for ‘unhealthy’ weight-for-height indices less than the 0.135^th^ (−3 SD) or greater than the 97.7^th^ (+2 SD) centiles, in order to achieve a “non-obese sample with equal height” [5]. Importantly, the resulting reference charts align much better at age 19y with the usual adult definitions of overweight (BMI >25 kg/m^2^) and obesity (BMI >30 kg/m^2^), which correspond to WHO BMI centiles 85 (+1 SD) and 97 (+2 SD) [5].

CPEG acknowledges the rigorous standards for data collection and analysis used by the WHO and promotes these new growth charts with emphasis on the use of BMI. Nevertheless, in a recent survey by the Canadian Paediatric Society, almost half (49.7%) of chart users cited the lack of weight-for-age reference for older children as a hindrance [12]. This is particularly true for health-care workers following children with systemic illnesses, where weight loss may reflect disease activity even before it impacts on statural growth [22,23]. In both infants and older children, correct interpretation of BMI changes also requires concurrent inspection of height and weight-for-age [24-26]. In addition, weight-for-age z-scores are important for both individual and population screening, especially in the assessment of underweight, where weight-for-age z-scores are an independent predictor of mortality and an important clinical research tool [1].

To address these user concerns [10-13], we have generated complementary charts that enhance the clinical utility of the WHO norms. Importantly, we have retained the WHO results where available for height-for-age, BMI-for-age (≥2y), weight-for-age (0–10y), length-for-age, weight-for-height, and head circumference-for-age (0–2y); for these curves, we have used published WHO LMS parameters to reformat the WHO charts to reflect the traditional CDC choice of centiles.

Ideally, any extension to the weight-for-age centiles would be based on the same data used to construct the WHO reference charts (i.e. the NCHS data *and* the cross-sectional portion of the MGRS). Nevertheless, the extension of weight-for-age in our revised charts provides a plausible alternative that can be used by health-care workers interested in simultaneously charting weight and height in older children. While we did not have access to ~ 8000 observations from the WHO MGRS (aged 18–71 months), the influence of these missing data is clear from the comparison of the smoothed NCHS-R and WHO weight-for-age centiles in Figure 2 and Table 1. In general, the two sets of curves align well for boys at all centiles and for girls at the median and lower centiles, which speaks to a rightward skew and higher weight-for-age in the North American data for girls. Although this trend is known to be worsening over time [21,27], it is clearly an issue even in the earlier period 1963–75. Regardless of these modest differences, we must remember that the extended weight-for-age charts are intended only as a reference, a yardstick against which growth may be measured [2,5]. In this, they are intended to complement — rather than supplant — existing WHO charts, and the definition of obesity will continue to be based on WHO BMI norms.

WHO charts routinely display curves at 1, 2 and 3 SD (z-scores) above and below the median, (i.e. centiles 0.1, 3, 15, 50, 85, 97 and 99.9), which results in fewer lines between the 3^rd^ and 97^th^ centiles. The bootstrap results suggest that the 99.9^th^ centiles for weight and BMI should be interpreted with caution. With a non-normal distribution, these outlying centiles — each representing ~1/1000 children — are simply difficult to estimate precisely with only 673 ± 204 (mean ± SD) boys/yearly interval and 646 ± 185 girls/yearly interval between 5–19y (NCHS-R). To address these concerns, our new charts remove the 0.1 and 99.9 centile lines. Instead, we implement the more familiar CDC choice of centiles (3, 10, 25, 50, 75, 90 and 97 for height and weight and 3, 10, 25, 50, 85, and 97 for BMI), to provide a more granular description within the normal range and to more easily detect aberrations in growth at an earlier stage. This is particularly important for weight-for-age, where failure-to-thrive is often defined by crossing 2 centile lines [23].

## Conclusion

In summary, we have extended weight-for-age beyond 10y using the same core data used to create the WHO reference for school-aged children and adolescents. Moreover, the reference population was trimmed using the same exclusion criteria as the WHO reference, and optimal smoothing models were identified and confirmed using the same diagnostic criteria [4,5]. The smoothed centiles capture the empiric centiles well for all levels 3–97%. We believe that our revised pediatric growth charts will dovetail with the existing WHO charts to provide a full range of growth charts. The CPEG charts complement existing WHO charts by allowing weight-for-age to be plotted concurrently with height in older children. The additional centiles in the normal range will also allow for more precise assessment of normal growth and earlier detection of failure-to-thrive as weight-for-age crosses centiles. A complete set of charts is freely available at the CPEG web site (http://cpeg-gcep.net) along with an assortment of useful spreadsheet tools for calculating centiles and z-scores from clinical data.

## Abbreviations

BCPE: Box-Cox power exponential model; Parameters: λ: power transform of abscissa; μ: Median (M); σ: Coefficient of variation (S); ν: Skew exponent (L); τ: Kurtosis; BMI: Body Mass Index; df: degrees of freedom; CDC: Centers for Disease Control and Prevention; CPEG: Canadian Pediatric Endocrine Group; LMS: BCPE model in the absence of kurtosis (τ = 2); kg: kilograms; MGRS: WHO Multicentre Growth Reference Study; NCHS: National Center for Health Statistics; NCHS-R: N = 22,917 NCHS dataset after exclusion of 314 girls, 304 boys; SD: standard deviation; WHO: World Health Organization; y: Years.

## Competing interests

The authors declare that they have no competing interests.

## Authors’ contributions

The 3 primary authors were members of the ‘Methodology Subcommittee’ of the CPEG Working Committee for National Growth Charts. In this capacity, all 3 participated in selection of appropriate data preparation and statistical methodologies. AS performed these statistical analyses, prepared the reformatted growth charts, and contributed to the drafting and revision of the manuscript. CR and DM participated in the design of the new growth charts, including the selection of centiles and layout for the new charts, and contributed to the drafting and revision of the manuscript. All authors read and approved the final manuscript.

## Authors’ information

As members of the national working committee sponsoring the development of these new charts, EC, SL, JPC and MP all participated in their design, including the selection of centiles and layout for the new charts, and they assisted with the revision of the manuscript.

## Pre-publication history

The pre-publication history for this paper can be accessed here:

http://www.biomedcentral.com/1471-2431/14/32/prepub

## Supplementary Material

Additional file 1: Table S1 Descriptive statistics for age and gender cohorts: Boys (A) and Girls (B). **Table S2.** Mean absolute discrepancy (MAD, kg) vs CDC 2000 curves. Click here for file
