# Supplementary material for: Extending World Health Organization weight-for-age reference curves to older children
Source: BMC Pediatr. 2014 Feb 3;14:32. doi: 10.1186/1471-2431-14-32 (PMC3922078; doi:10.1186/1471-2431-14-32)
Supplement: Additional file 1: Table S1 — Descriptive statistics for age and gender cohorts: Boys (A) and Girls (B). Table S2. Mean absolute discrepancy (MAD, kg) vs CDC 2000 curves. [file 1471-2431-14-32-S1.pdf]

Table S1: Descriptive statistics for age and gender cohorts: Boys (A) and Girls (B)

Boys Weight								
Age	10-11	11-12	12-13	13-14	14-15	15-16	16-17	17-18
Q1	29.3	33.0	36.1	41.7	48.5	53.3	58.1	60.3
Median	32.7	36.7	41.3	48.1	54.8	59.9	63.7	65.5
Mean	33.7	38.1	42.6	49.4	55.8	60.9	64.7	67.3
Q3	36.5	41.4	47.6	55.6	62.4	66.9	70.4	73.3
SD	6.32	7.54	9.07	10.70	10.69	10.94	10.23	10.46
Skew	1.22	1.30	0.78	0.86	0.45	0.85	0.61	0.73
Kurtosis	2.46	2.91	0.51	1.14	0	1.63	0.74	1
N	744	747	861	779	776	766	714	613

Girls Weight								
Age	10-11	11-12	12-13	13-14	14-15	15-16	16-17	17-18
Q1	29	33.6	39.2	43.1	47.2	48.8	49.7	50.8
Median	33.4	38.3	45.4	49	52	54.3	54.9	56
Mean	34.7	40.1	46.5	50.4	53.6	55.5	56.5	56.9
Q3	38.6	45.1	51.8	56.2	59.3	59.9	61.5	61.9
SD	7.44	9.13	9.98	10.18	9.35	9.75	9.81	9.12
Skew	1.00	0.86	0.72	0.81	0.66	0.94	1.08	0.88
Kurtosis	1.63	0.55	0.77	1.14	0.55	1.22	1.58	1.59
N	759	683	747	780	748	659	684	582

Descriptive statistics for weight (kg) by age and gender for subjects in the NCHS-R dataset (after exclusions). Interquartile range is given by Q1-Q3. A symmetric distribution will have skew = 0 and a normally peaked distribution will have sample kurtosis = 3 (not to be confused with the τ parameter in the BCPE model, as discussed in the statistical methods manual [17] available at http://www.cpeg-gcep.net)

Table S2: Mean absolute discrepancy (MAD, kg) vs CDC 2000 curves

Smoothed Centiles	Boys 5-19y MAD	Girls 5-19y MAD
3	0.47±0.35	0.52±0.34
25	0.58±0.40	0.64±0.41
50	0.64±0.41	0.78±0.45
75	0.84±0.54	0.93±0.41
97	3.48±1.55	4.35±3.14

Mean absolute discrepancy (MAD, kg ± SD) is based on monthly observations from NCHS-R and CDC 2000 weight-for-age reference curves (5–19y).
